# Barriers and Facilitators to Integrating Family Caregivers Into the Healthcare Team

**DOI:** 10.1093/geroni/igaa057.2411

**Published:** 2020-12-16

**Authors:** Esther Friedman, Patricia Tong

**Affiliations:** RAND, santa monica, California, United States

## Abstract

Recent changes in health care practices - shorter hospital stays, increased complexity of disease management, and greater management of chronic illnesses at home - have left family members increasingly responsible for medical tasks. Although these family caregivers represent the vital front line of care, they remain mostly excluded from the formal health care team. Based on interviews with multi-sector stakeholders representing patients/caregivers, providers, and payers, we identified several barriers to fully incorporating family caregivers into the health care team. They fall under five key themes: identification of caregivers; communication and information; time and resources; trust and cultural barriers; and Medicaid coverage. We discuss these barriers and potential solutions that could be undertaken by different stakeholder groups to improve family caregiver integration into the care team.

